# The impact of the COVID-19 pandemic on Polish orthopedics, in particular on the level of stress among orthopedic surgeons and the education process

**DOI:** 10.1371/journal.pone.0257289

**Published:** 2021-09-24

**Authors:** Łukasz Kołodziej, Dawid Ciechanowicz, Hubert Rola, Szymon Wołyński, Hanna Wawrzyniak, Kamila Rydzewska, Konrad Podsiadło

**Affiliations:** 1 Department of Orthopaedics, Traumatology and Orthopaedic Oncology, Pomeranian Medical University of Szczecin, Szczecin, Poland; 2 Department of Clinical and Molecular Biochemistry, Pomeranian Medical University of Szczecin, Szczecin, Poland; China University of Mining and Technology, CHINA

## Abstract

The Coronovirus Disease 2019 –(COVID-19) pandemic had a significant impact on the health care system and medical staff around the world. The orthopedic units were also subject to new restrictions and regulations. Therefore, the aim of our research was to assess how the COVID-19 pandemic affected orthopedic wards in the last year in Poland. We created an online survey, which was sent to 273 members of the Polish Society of Orthopedics and Traumatology. The survey contained 51 questions and was divided into main sections: Preparedness, Training, Stress, Reduction, Awareness. A total of 80 responses to the survey were obtained. In Preparedness section the vast majority of respondents (90%) replied, that they used personal protective equipment during the pandemic, however only 50% of the respondents indicated that their facility received a sufficient amount of personal protective equipment. Most of the respondents indicated that the pandemic negatively affected the quality of training of future orthopedists (69.4%) and that pandemic has had a negative impact on their operating skills (66,7%). In Reduction section most of the doctors indicated that the number of patients hospitalized in their departments decreased by 20–60% (61,2% respondents), while the number of operations performed decreased by 60–100% (60% respondents). The negative impact of pandemic on education was noticeable especially in the group of young orthopedic surgeons: 0–5 years of work experience (p = 0,029). Among the respondents, the level of stress increased over the last year from 4.8 to 6.9 (p <0.001). The greatest increase in the level of stress was observed among orthopedists working in country hospitals (p = 0,03). In section Awareness 36,3% of respondents feel well or very well informed about the latest Covid-19 regulations. In addition, most doctors (82.6%) believe that the Polish health care system was not well prepared to fight the pandemic and that the regulations applied so far are not sufficient to effectively fight the pandemic (66.2%). The COVID-19 pandemic has impact on orthopedics departments in Poland and negatively affected the quality of training of orthopedic surgeons and the level of stress.

## Introduction

In December 2019 several cases of pneumonia caused by an unknown pathogen were reported by the Chinese government. A new variant of coronavirus was identified in infected patients with the ability to cause severe acute respiratory disease and named SARS-CoV-2, however a disease caused by the virus was named Covid-19 (Coronavirus disease 2019) [1, 2]. The uncontrolled spread of SARS-CoV-2 was declared a global pandemic on 11.03.20 by WHO [3, 4]. The first case in Poland was documented on 4.03.2020 and shortly afterward first governmental restrictions came into life [5]. Involving a wide range of fluctuation in the realm of public life, they were supposed to restrict the spread of the pathogen. Set changes had a significant impact on public healthcare.

The main assumption was to maximize the capacity of intensive care units and to provide the staff with the necessary personal protective equipment. The changes aimed at preparing for admitting to hospitals an uncommonly sizable number of Covid-19 patients and at mobilizing as many health service workers as possible. The transformations also involved advances in elective and trauma surgeries and other therapeutic and diagnostic treatment including long-term hospitalization that has been indicated in our studies.

Despite the available studies on the impact of the pandemic on the health care system in the world, there are still few reports of how the pandemic and government regulations have affected orthopedic wards. Additionally, the available publications do not directly refer to the level of stress among orthopedic doctors and the process of specialization training for residents, and further education of doctors [6–9]. Therefore, our research was aimed at identifying the current challenges that orthopedics in Poland is facing in the present circumstances. Its purpose was to identify the nature of contemporary impediments directly underlining the stress experienced in practice, the changes in surgeries, access to operating rooms and medical supplies, and finally form of encounter with the patient. Likewise, the impact of SARS-CoV-2 had on the process of resident doctors’ training and further specialists’ education was analyzed. All that in the aim of quicker identification and efficiency in dealing with the issue in a possible future crisis.

## Materials and methods

### Participants

In the study, an online-based anonymous voluntary survey was conducted within the Polish Society of Orthopedics and Traumatology (PTOiTR). Between February 15^th^, 2021, and March 15^th^, 2021, to 273 participants were sent an online questionnaire. Data was colected in two voivodeships: West Pomeranian and Malopolskie.

### Questionnaire

In the study, an online-based anonymous voluntary survey [S1, S2 Files] was created and uploaded on the Google online survey platform. For the study, we designed a questionnaire in Polish language, as well as an English translation, are appended as supplementary material. The link to the survey was sent to the members of PTOiTr in the form of an e-mail with an invitation to participate in the study and a description of the objectives of the study. The survey was mainly based on a published study by Randau T.M. et al. and others similar publications, adjusting the questions to the situation in Poland [6–9]. In addition, the survey was to examine more closely the level of stress among orthopedists in Poland and the impact of the pandemic on the process of training orthopedists. For this purpose, questions 47 and 48 were added, in which the respondents had to sequentially assess their stress level before the pandemic and now. Questions about training were also added to the survey, where the main assumption was to check how respondents assess the effects of the pandemic on the level of training and whether they are willing to use online forms of learning. The questionnaire was divided into several main domains (5): Preparedness (questions 7–15); Training (16–26); Stress (27–33); Reduction (34–41); Awareness (42–45+49,50) and single questions–Stress level and Financial problems. Aiming at minimizing the prospect of suggested answers, in blocks 1., 2., 3. and 5. that focused on a subjective participants’ evaluation, a Likert-like scale was applied (from 1 to 5), in which the surveyed could choose to what degree they fully did not agree (1) or did fully agree (5) with a given thesis (only block 3., concerning the stress levels, a 1–10 graded scale was applied, in which 1 indicated significantly low and 10 significantly high levels of the studied parameter). In block 4. that focused on the amount of hospitalized patients, undertaken procedures and interventions, a 5-grade scale (80–100%, 60–80%, 40–60%, 20–40%, 0–20%) with a “hard to determine” answer option was applied. It facilitated an estimated percentage decline in the studied parameters.

### Statistics

The responses from the survey were collected into a separate file in Microsoft Excel 2019. The correctness of the entered data with the survey responses was checked by 4 study authors (DC, HR, HW, SW). Data quality control was performed by checking skewness and kurtosis for normality in the questions on a five-point response scale. We performed a descriptive analysis, calculating the mean, standard deviation, standard error, and 95% confidence intervals for all numerical questions. We performed a correlation matrix analysis by computing Spearman’s R between all numeric questions and domain. Finally, we performed the Wilcoxon paired data to test and the Kruskal-Wallis test between domains and selected general questions. Significant level was set at p<0.05.

### Ethical approval and participans consent

Ethical approval was obtained from the Bioethics Committee at the Pomeranian Medical University in Szczecin (Approval number: KB-0012/55/04/2021/Z).

In email messeges, survey participants were assured all data would be used only for research purposes and data set will not be available for public. Participants’ answers were anonymous and confidential according to Google’s privacy policy. Participants did not have to mention their names or contact information. In addition, participants could stop participating in the study and could leave the questionnaire at any stage before the submission process and their responses were not saved. Response were saved only by clicking on “submit” button. By completing the survey, participants were acknowledging the above approval form and were consenting to voluntarily participate in this anonymous study.

## Results

### Demographic characteristics

**Fig 1:**
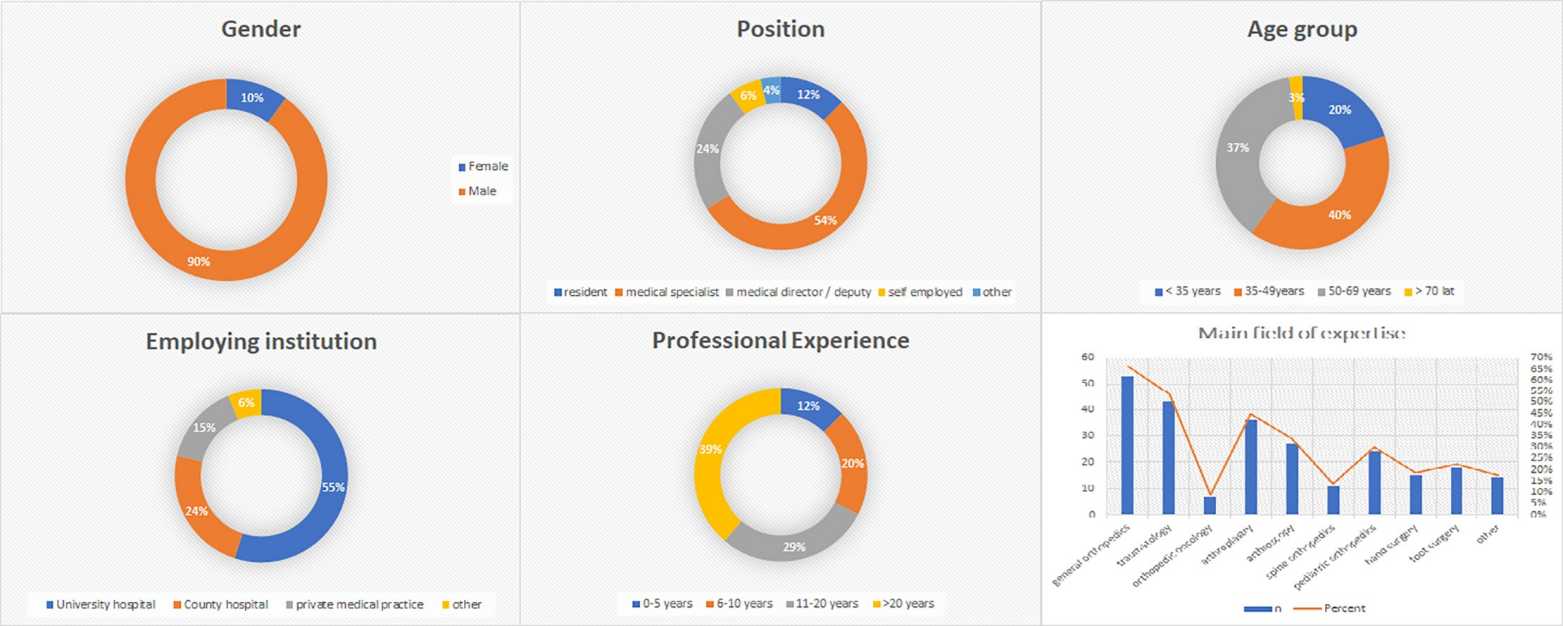
General description of the study group. The graphs show the composition of the participants’ profile information. Summarizes gender, position, age, employing institution, professional experience, and main field of experience.

A total number of 80 responses were collected, thus achieving a 29% response rate. Most of respondents were male (n = 72, 90%). Two age groups were the most numerous: 35–49 years (n = 32, 40%) and 50–69 years (n = 30, 37,5%). Mostly the respondents indicated that they had been working in the profession for over 20 years (n = 31, 38,7%) and that the most numerous group were doctors working in a university hospital (n = 44, 55%). All demographic results are shown in Fig 1 [Fig 1].

### Preparedness

The purpose of the questions in this domain was to check how the respondents assess the hospital’s preparedness to fight the pandemic and what conditions prevailed in the orthopedic wards during the pandemic. A smaller proportion of respondents (40%, n = 32) stated that their main workplace was directly involved in the treatment of COVID-19 patients. This may mean that a smaller proportion of orthopedic wards have been renamed COVID-19 wards. Almost all respondents (90%, n = 72) that their workplace has implemented protective measures and a change in work organization to counteract the spread of COVID-19. Almost all respondents (90%, n = 72) that their workplace had implemented protective measures and a change in work organization to counter the spread of COVID-19, however only 38% (n = 30) of respondents reported that staff had been divided into smaller groups to minimize the risk of infections. Additionally, only half of the respondents (n = 40) indicated in the questionnaire that their ward received a sufficient amount of personal protective equipment (disinfectants, gowns, gloves, FFP2 / FFP3 masks) In question 10 on the isolation of patients infected with SARS-CoV-2, approximately 69% (n = 55) of the respondents indicated that in their practice, infected patients are isolated from uninfected patients. About 69% (n = 55) reported problems with staff shortages resulting from infecting staff members and quarantines. About 39% of the surveyed doctors (n = 31) used telemedicine in their practice (telephone consultations), however only about 21% (n = 17) believe that telemedicine should be used in the future.

#### Training

In question number 16 of the Training domain respondents were asked about impact COVID-19 pandemic on number of performed operations by them and the majority indicated that the amount of surgeries were decreased during the pandemic (n = 41, 65.1%). In addition, as many as 50% of doctors in question number 17 concerning impact pandemic on efficiency of specialists’ training claim that the pandemic negatively affected the quality of training of future orthopedists (n = 43, 69.4%). Most doctors (n = 40, 66.7%) also believe that the pandemic has had a negative impact on their operating skills. Despite the lack of access to face-to-face conferences, which was shown in questions 22 regarding attendance to conferences, in question number 23 and 24 about usage of online learning methods, most respondents used online conferences (n = 42, 67.7%) and webinars (n = 40, 65.5%). However, only 38.7% (n = 24) of respondents believe that online learning has had a positive effect on their knowledge and skills. The Kruskall-Wallis analysis shows significant differences of the influence of professional experience on the Training domain where we can see that the highest mean has a group with 0–5 years experience and the lowest group with over 20 years experience (p = 0,029) [Table 1].

**Table 1:** Influence of professional experience on the training domain.

Professional experience	N = 80	Mean (SD)	SE	95% Cl		P value
0–5 years	10	0,79	0,02	0,721823	0,858177	
		(0,11)				
6–10 years	16	0,73	0,03	0,653176	0,806824	0,02911
		(0,13)				
11–20 years	23	0,67	0,04	0,611201	0,728799	
		(0,12)				
>20 years	31	0,64	0,03	0,569878	0,710122	
		(0,16)				

Descriptive analysis and data quality, by a number of participant (N), mean, standard deviation (SD), standard error of the mean (SE), 95% confidence interval (95% CI)

### Stress level

We conducted paired stress analysis before March 2020 and after [Table 2]. Responders in questions 47 and 48 were asked about their feelings about stress in workplace before Pandemic and now. We can see that the level of stress is lower before the pandemic starts. The level of stress after March 2020 is higher by almost 2 points (p<0.001). The Kruskal-Wallis test shows significant differences by the analysis of the influence of the place of employment on the Stress domain. It shows significant differences where we can see that the highest mean on the stress level has a group working in County hospitals and the lowest mean has a group working in other places (p = 0,03) [Table 3]. Additionally, we wanted to check which factors were stressful for the greatest number of respondents. According to question 27, approximately 62% of respondents (n = 52) do not feel safe performing a physical examination of a patient suffering from COVID-19. In questions 29 and 30, the respondents were asked about the fear of being infected with Sars-COV-2 virus and about 63% of respondents (n = 50) report fear of being infected in the workplace, while about 74% of respondents (n = 59) feel anxious about greater risk of infection of family members. Half of the respondents (n = 40) feel a greater workload today than before the Pandemic. This fact may result from the greater amount of on-duty work at the hospital, as approximately 51% of the respondents (n = 41) in question 32 report that due to staff shortages, it was necessary to undertake more shifts at the hospital. Additionally, about 84% (n = 67) of respondents marked that the use of additional safety measures makes it more difficult to work in an orthopedic ward. It is worth emphasizing, however, that the respondents do not believe that the pandemic has a negative impact on the friendly relations between medical staff. In question 33, approximately 28% of respondents (n = 22) reported a deterioration of relations between staff.

**Table 2:** Level of stress before March 2020 and after (Willcox test). Descriptive analysis and data quality, by a number of participant (N), mean, standard deviation (SD), standard error of the mean (SE), 95% confidence interval (95% CI).

Level of Stress	Mean (SD)	SE	95% Cl		P value
Before March 2020	4,8125 (2,025744)	0,226485	4,368597	5,256403	
					1.302e-10
After March 2020	6,8875 (2,244508)	0,250944	6,39566	7,37934	

Descriptive analysis and data quality, by a number of participant (N), mean, standard deviation (SD), standard error of the mean (SE), 95% confidence interval (95% CI)

**Table 3:** Influence of employing institution on the stress domain.

Employing institution	N = 80	Mean (SD)	SE	95% Cl		P value
University hospital	44	0,68	0,02	0,641588	0,718412	
		(0,13)				
County hospital	19	0,71	0,03	0,660539	0,759461	0,032
		(0,11)				
Private medical practice	12	0,63	0,04	0,550789	0,709211	
		(0,14)				
Other	5	0,54	0,03	0,478643	0,601357	
		(0,07)				

Descriptive analysis and data quality, by a number of participant (N), mean, standard deviation (SD), standard error of the mean (SE), 95% confidence interval (95% CI).

### Reduction

The purpose of the questions in this domain was to check how, according to the respondents, the number of patients and procedures performed in the orthopedic ward changed. According to 61% of orthopedists (n = 49), the number of patients in the orthopedic department decreased by about 20–60%, however, the number of emergency cases decreased by about 0–40%, according to 58% of respondents (n = 46). The number of surgeries performed during the pandemic decreased by 60–100% according to 40% of respondents (n = 32) and by 20–60% according to 38% of the respondents (n = 30). The number of emergency operations performed due to injuries decreased by 20–40% according to 58% of the respondents (n = 46). It is worth emphasizing, however, that according to approximately 66% of orthopedic surgeons, the number of patients who canceled the scheduled visit / surgery themselves was 0–40%.

### Awerness and single questions

The goal of this domain was to find out how orthopedists evaluate the government’s actions to fight the COVID-19 pandemic and how well they feel informed about the pandemic. The vast majority of respondents (83%, n = 66) indicated that the Polish health care system is not well prepared to fight the pandemic and that the regulations applied so far are not sufficient to effectively fight the pandemic (66%, n = 53). In question 42, we checked how well-informed orthopedists feel about the new regulations to fight the COVID-19 pandemic and about 36% of respondents (n = 29) feel well-informed, 29% (n = 23) feel poorly informed, while 35% (n = 28) assessed that they felt neither well nor badly informed.

Finally, we asked questions not directly related to any of the domains listed above. In question 49, about 44% of orthopedic surgeons (n = 35) believe that their work regime will begin to return to before-pandemic state in the coming months and 83% of respondents (n = 66) believe that they will no longer be transferred to other departments due to staff shortages. Additionally, only 30% of respondents (n = 24) reported that the pandemic had a negative impact on their finances.

### Questionnaire—statistics

**Fig 2:**
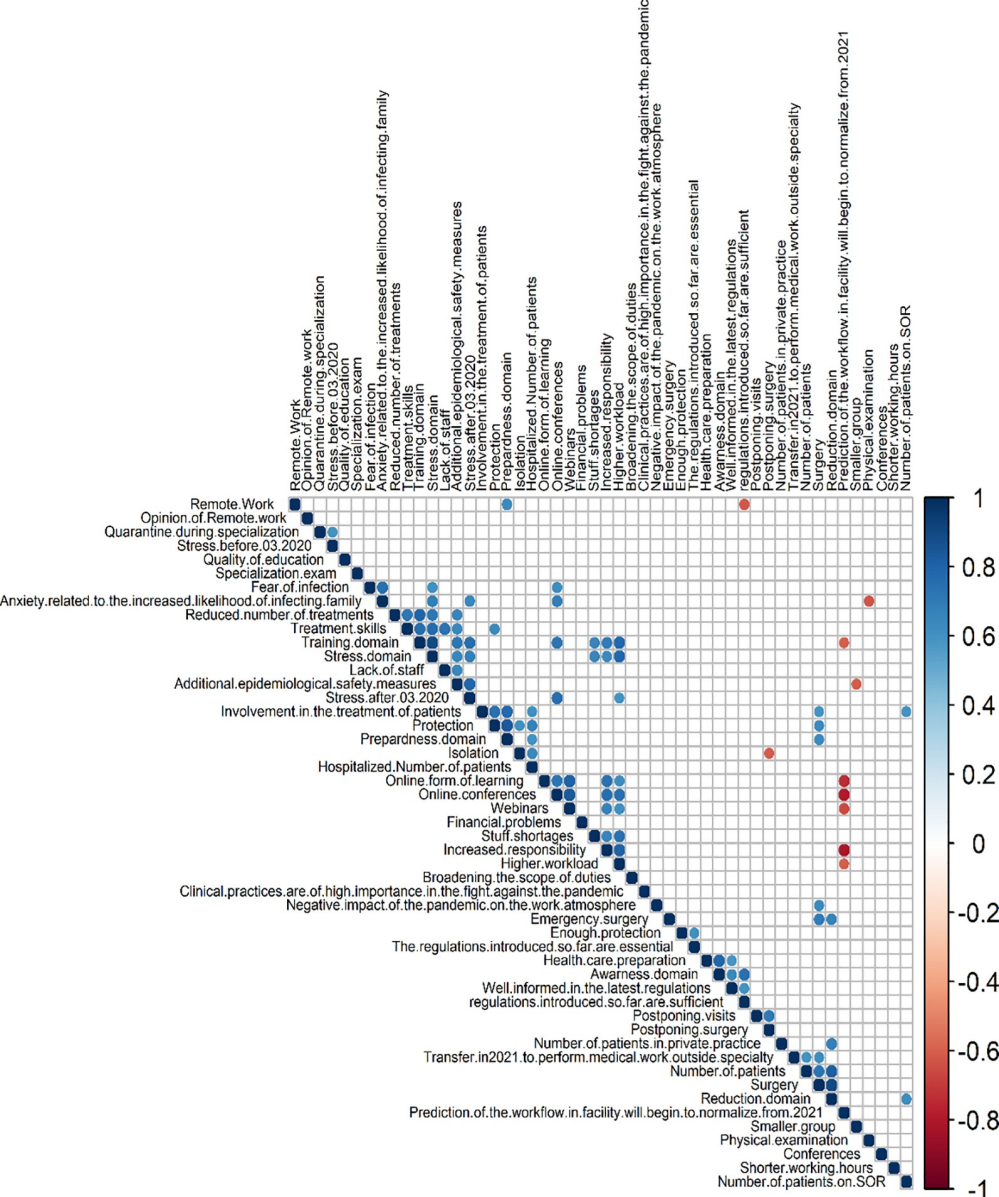
Correlation matrix. The figure shows a map of the Spearman correlation between the different profile items and the calculated indices. Red boxes presented a negative correlation (Spearman *r* < 0), blue boxes presented a positive correlation (Spearman *r* < 0), blue boxes presented a positive correlation (Spearman *r* > 0), darker colors represent stronger correlation, and white or light-colored boxes no or weak correlation.

Skewness ranged from over -1 to over 1. The highest value of kurtosis is over 4.5 and the lowest -1.5, thus the spread of data is considerable summarizes the data quality control. The Likert scale has been converted to numerical values, where 1 is the answer in full disagreement and 5 in full agreement. From this, the mean, standard deviation (SD), median, standard error(SE) and confidence interval(95% CI) could be calculated for example “Protection” has an average score of 4,55 and standard deviation of 0,76, which means that most of the answer on the question “My office / my clinic / I have implemented protection measures and a change in work organization to prevent the spread of COVID-19.” was fully agree and most of the answers were the same. The main domains were calculated as a percentage of the maximum number of points (for example “ss” has a score of 0,63 which means that is 63% of the maximum score for each question). Next, we conducted a descriptive analysis of all profile questions, summarizing the results. The first figure depicts all answers to the question related to Covid-19. Descriptive analysis on (1) the impact of the pandemic on your office/clinic/institution, (2) training, (3) the impact of the pandemic on stress and overwork, (4) the impact of the pandemic on daily work and patients, (5) assessment of measures taken by the government and influence on society and prospects. The distribution of responses to the survey questions is presented in the Figure attached as a supplement to the publication [S1 Fig]. We also conducted the correlation analysis among the index questions. The results are presented in Fig 2 [Fig 2].

## Discussion

From the very beginning of the COVID 19 pandemic in Poland, it was necessary to introduce changes, such as—additional personal protective equipment, reorganization of the distribution of patients, and change of doctors’ work schedules. In our study group, the vast majority of doctors (90%) used personal protective equipment during the pandemic to prevent the spread of the Sars-Cov-2 virus. However, it is worth emphasizing here that only 50% of the respondents indicated that their facility received a sufficient amount of personal protective equipment and that they were properly protected during work. This may indicate that there are still problems with the availability of personal protective equipment. Coelho da Maina et. all show that the problem of the lack of personal protective equipment among health care workers also applies to other countries in the world [10]. Additionally, in our study around 36% of respondents feel well or very well informed about the latest Covid-19 regulations. However, most doctors (82.6%) believe that the Polish health care system was not well prepared to fight the pandemic and that the regulations applied so far are not sufficient to effectively fight the pandemic (66.2%). For comparison, Randau T.M. et al. have shown, using the example of the orthopedic population in Germany, that over 71% of them feel well informed about the new COVID-19 regulations and that around 81% believe that government regulations are necessary. However, only 68% of orthopedic surgeons in Germany believe that the regulations introduced are sufficient to fight the pandemic [6]. The above data may indicate that doctors are being informed too late or insufficiently about new regulations in the fight against the COVID-19 pandemic in Poland.

As the available publications show, the COVID 19 pandemic negatively affected the mental health of health care workers [11–13]. Our research shows this as well. Among the orthopaedic surgeons surveyed, the stress level increased from 4.8 before March 2020 to 6.8 points now. Thus, the stress level during the pandemic year increased by an average of more than 2 points. There may be several reasons for this. Elbay et. all shows that levels of depression and stress among healthcare professionals can be affected by increased workload and less support from colleagues [14]. In our group, 69% of respondents indicated that their facility was struggling with staff shortages. In addition, about 50% of respondents indicated that they felt a greater workload during the pandemic and that due to staff shortages it was necessary to undertake more shifts than before March 2020. Another factor that could have contributed to a greater feeling of stress among doctors is fear for their health and the health of their families [15]. This is also shown in our study, where 63% expressed fear of infection with the SARS-CoV-2 virus, and 74% of respondents confirmed that they feel fear of infecting their family members. As it can be suspected, district hospitals and smaller treatment units are struggling with the greatest shortages of staff and, consequently, the greatest increase in the level of stress among doctors during the COVID-19 pandemic. This is confirmed by our study, where significant statistical differences were shown for the impact of the place of employment on the stress level. The highest increase in the level of stress was observed in the group of doctors working in country hospitals. It can also be assumed that the greater level of stress among doctors working in these hospitals could also result from the lack of personal protective equipment.

After correlation analysis, we made a matrix of Spearman correlation between questions and domain against each other. There were some significant results. Question about prediction workflow to normal correlated negatively with questions regarding online form of teaching, online conferences, increased responsibility, and higher workload–they were mostly affirmatively answered by resident physicians. In concluding young doctors are skeptical about the chances that work at a hospital will come back to normal in 2021. Only 44% of the respondents believe that the work in their facility will start to normalise from 2021. This condition is similar to the situation in other countries in Europe [16, 17]. Public health analysts are indicated that this state could not change to the pre-COVID era [18]. The next noticeable positive correlation is between domain Training, domain Stress, and questions about staff shortage, higher workload, and increased responsibility. Healthcare workers during pandemics suffer from mental health disorders, which can be caused by work at first line with COVID-19 pandemic [19]. It only proves that the state of emergency caused by COVID-19 is making a more stressful environment for healthcare personnel. It is worth a mention the interesting negative correlation between the question about remote work and the question about sufficient introduced regulations. Supporters of remote work think that regulations are insufficient. This opinion is present among European countries [20–22]. Last but not least worth mention is the positive correlation between Treatment skills and the Training domain, Stress domain, lack of staff, and additional epidemiological safety measures. This interdependence shows how great impact the stress related to the COVID-19 pandemic has on training and medical skills, thereby the future of orthopaedic surgery. In the USA orthopaedic specialization course is longer than in Poland, thus it can be more affected by unusual circumstances as one of the trainees describe [23].

Results of Kruskal-Wallis analysis show inversely proportional professional experience on Impact on the training domain. The highest mean is assigned to the group with the lowest experience of 0–5 years and the lowest mean is assigned to a group with experience of over 20 years. It shows that the effects of pandemic impact the most on the training process of the young, inexperienced surgeons, which lead us to the higher stress level. This trend can be seen in other parts of the world, like India [24]. There also occurred issue concerning lack of possibilities to train young residents. The more experienced surgeon, the significantly lesser the mean in the Training domain, which can be seen in groups 6–10 years and 11–20 years of experience [23]. One of the most important in work of the orthopedic surgeon is manual skills as well as physical examination. Nowadays physical examination can be somehow replaced with telemedicine usage, as can be seen in the USA, still, surgical skills cannot be succeeded as online version [25]. With canceled elective operations, few acute surgeries, and quarantine time out of hospital grows the fear about the wave of new, not enough educated physicians. A similar situation seems to be in the United Kingdom, where consequences can be observed on every level of training structure [26]. The process of repairing the healthcare field will take a great amount of time and effort. It should be taken into consideration additional training programs or change the structure of orthopedic wards to involve more residents physicians in surgeries. It is also possible to train on cadavers but the opinions are divided, especially in time of the COVID-19 pandemic [27–29]. With the still-going progress of the IT-sector, we have a new opportunity to try new technology like surgical simulators or VR reality, as a replacement for real-life surgery [27].

With lower experience, it can be observed higher overall stress levels than other responders. It correlates with reduces the number of surgeries, more responsibility on inexperienced residents, and additional safety measures like in Canada, the USA, or Malaysia [23, 30, 31]. Reduced number of elective surgeries and lockdown led to decrease surgical skills among residents, which have been spotted in USA trainees [23]. Although all medical conferences were canceled or changed into on-line meetings, the pandemic shows how users can be online teaching for example webinars or online conferences, but it cannot fully replace training in person. Results show that the main part of responders used webinars or attended online conferences like worldwide trend [24, 32, 33].

Our research has limitations that we are aware of. The first is the lack of validation of the online survey. However, the survey was based on a published study conducted in April 2020 [6]. Besides, we have modified the form in such a way as to be able to assess the impact of the COVID-19 pandemic on specialization training for residents and further education of specialist doctors. We also checked how the pandemic affected the level of stress in orthopedic doctors. Another limitation of our work is the relatively small number of respondents. However, after analyzing the demographic data, it can be seen that we have managed to reach orthopedists with different levels of experience and working in various places.

## Conclusion

Orthopedic departments in Poland during the last year have been struggling with many problems during the COVID-19 pandemic. One of the effects of these difficulties is the increase in stress levels among physicians. This is a problem that should not be underestimated and appropriate measures to reduce the level of stress should be introduced in the following months of the pandemic. Particular attention should be paid to the provision of an adequate amount of personal protective equipment, and proper training and information of medical personnel about the regulations introduced by the government to fight the COVID-19 pandemic. In addition, all procedures aimed at preparing the Polish health care system for further waves of SARS-CoV-2 infections should be implemented in order to avoid a further increase in the level of stress among orthopaedic surgeons. Difficulties in training orthopedists should also be taken into account. Taking into account the fact that the pandemic situation does not improve shortly, other training methods for young orthopedists are worth considering.

## Supporting information

S1 Fig Overview of the answers questions of the questionnaire. (PDF)

S1 File English version of the questionnaire. (PDF)

S2 File Polish version of the questionnaire. (PDF)

S1 Data (RAR)
