# Supplementary material for: The impact of the COVID-19 pandemic on Polish orthopedics, in particular on the level of stress among orthopedic surgeons and the education process
Source: PLoS One. 2021 Sep 24;16(9):e0257289. doi: 10.1371/journal.pone.0257289 (PMC8462693; doi:10.1371/journal.pone.0257289)
Supplement: S1 Fig — (PDF) [file pone.0257289.s001.pdf]

Prepardness domain

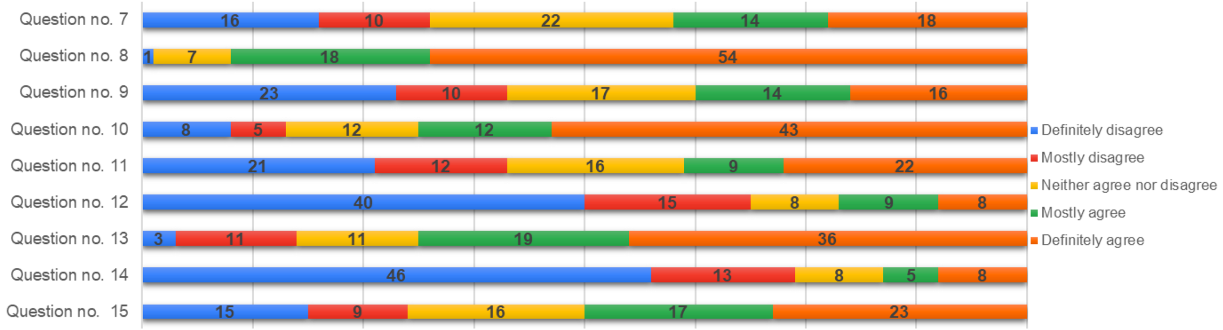

Training domain

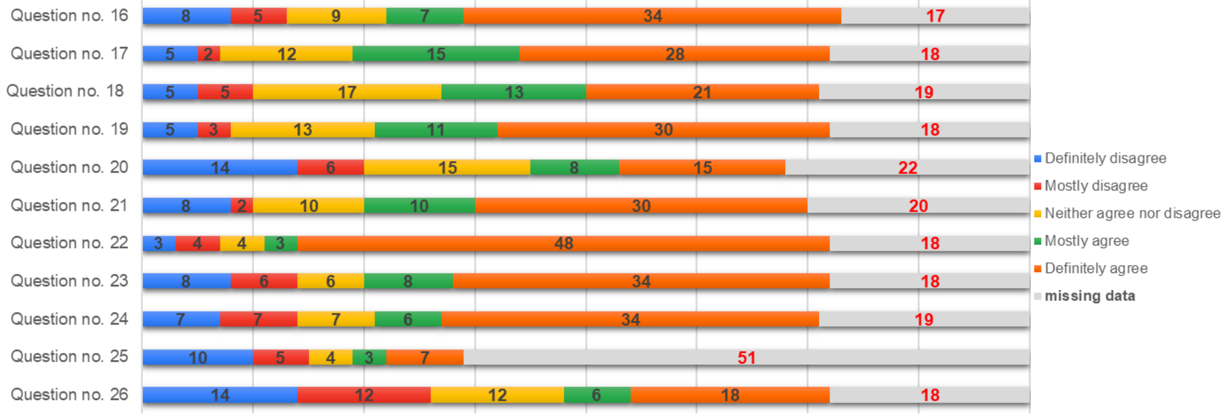

Stress domain

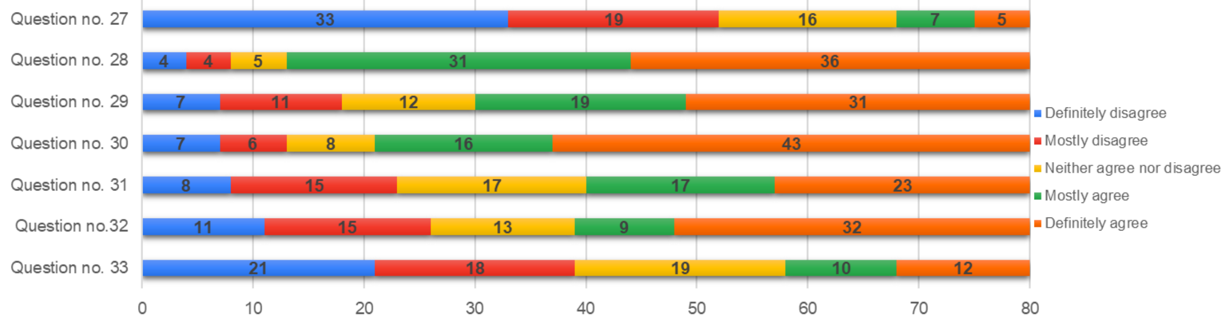

Reduction domain

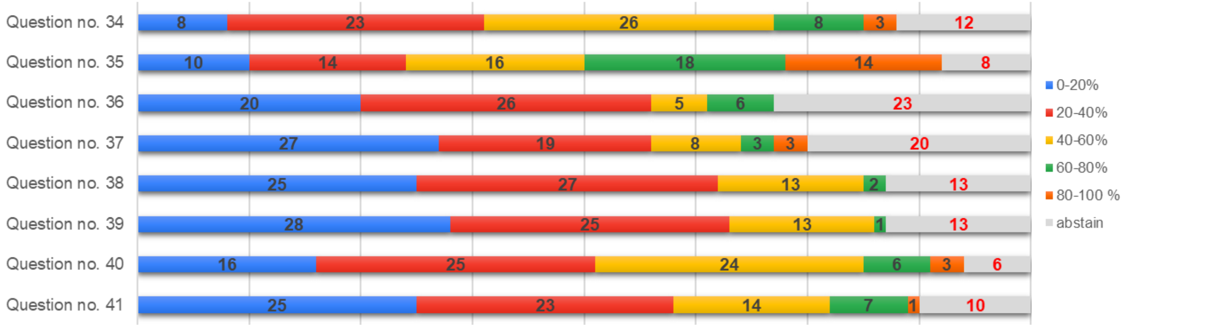

Awareness domain

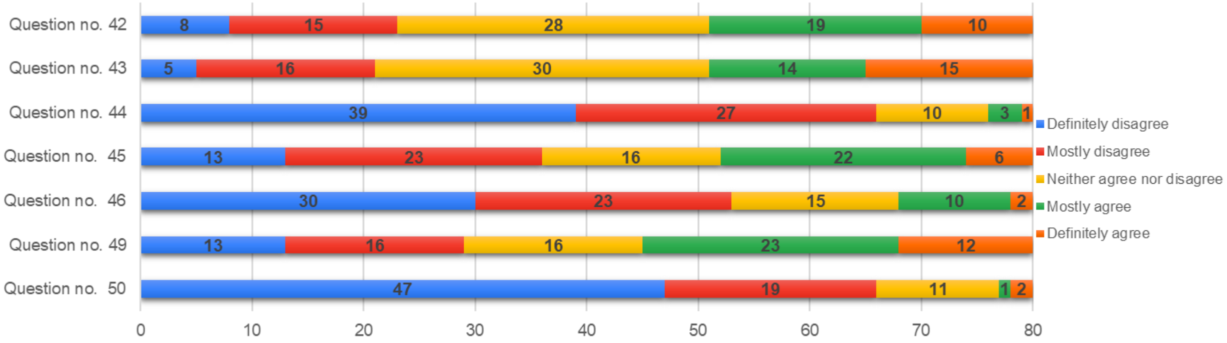

Overview of the answers questions of the questionnaire. The graph depicts the answers given by the participants. Designed to query certain dimensions of the pandemic impact, most of them contributing to one of five indices. Questions from 7 to 15 allow “definitely agree”, “definitely disagree” and “neutral” for an answer. Questions from 16 to 26 allowed “definitely agree”, “definitely disagree”, “neutral” and „no answer” marked with „missing data” for an answer. Questions from 27 to 33 allowed “definitely agree”, “definitely disagree” and “neutral” for an answer. Questions from 34 to 41 allowed “high percentage”, “low percentage”, “middle/ abstain” for an answer. Questions from 42 to 50 allowed “definitely agree”, “definitely disagree” and “neutral” for an answer.
