# Supplementary material for: The impact of the COVID-19 pandemic on Polish orthopedics, in particular on the level of stress among orthopedic surgeons and the education process
Source: PLoS One. 2021 Sep 24;16(9):e0257289. doi: 10.1371/journal.pone.0257289 (PMC8462693; doi:10.1371/journal.pone.0257289)
Supplement: S1 File — (PDF) [file pone.0257289.s002.pdf]

# The impact of the COVID-19 Pandemic on Polish Orthopedics

This is an anonymous survey to analyze the impact of the COVID-19 pandemic on specialist's work, trainee program stress level, and overwork. It contains 4 parts and will take about 10-15 minutes.

Thank you for your time and help!

I am aware that participation in the survey is voluntary and anonymous and I agree to participate in the survey by going to the next part of the survey

* Required

In this section we would like to know basic information about you.

1. 1) What is your gender? *

*Mark only one oval.*

- Female
- Male

2. 2) What is your age? *

*Mark only one oval.*

- < 35 years old
- 35-49 years old
- 50-69 years old
- > 70 years old

3. 3) How long have you been practicing as an orthopedic surgeon *

*Mark only one oval.*

- 0-5 years
- 6-10 years
- 11-20 years
- >20 years

4. 4) What position do you currently occupy in your main workplace? *

*Mark only one oval.*

- Resident physician
- Specialist
- Head of hospital ward
- Private practise
- Other

5. 5) Main field of interests: (more than 1 answer is possible) *

*Check all that apply.*

- [ ] General orthopedics
- [ ] Traumatology
- [ ] Musculoskeletal oncology
- [ ] Joints arthroplasty
- [ ] Arthroscopy and minimal invasive orthopedics
- [ ] Spine surgery
- [ ] Pediatric orthopedics
- [ ] Hand surgery
- [ ] Foot surgery
- [ ] Others

6. 6) In which kind of institution do you work currently? *

*Mark only one oval.*

- University Hospital
- County hospital
- Private medical practice
- Other

How the COVID-19 pandemic affect work in private practice/clinic/ward?

7. 7) My department/ my clinic/ I am currently involved in the treatment of COVID-19 positive patients. *

*Mark only one oval.*

| | 1 | 2 | 3 | 4 | 5 | |
|---|---|---|---|---|---|---|
| I completely disagree | | | | | | I fully agree |

8. 8) My office / my clinic / I have performed protection measures and a change in work organization to prevent the spread of COVID-19. *

*Mark only one oval.*

| | 1 | 2 | 3 | 4 | 5 | |
|---|---|---|---|---|---|---|
| I completely disagree | | | | | | I fully agree |

9. 9) On my ward, the staff has been divided into smaller groups to minimize the risk of contamination. *

*Mark only one oval.*

| | 1 | 2 | 3 | 4 | 5 | |
|---|---|---|---|---|---|---|
| I completely disagree | | | | | | I fully agree |

10. 10) ) On my ward, both healthy and infected patients were isolated in separate rooms. *

*Mark only one oval.*

| | 1 | 2 | 3 | 4 | 5 | |
|---|---|---|---|---|---|---|
| I completely disagree | ◯ | ◯ | ◯ | ◯ | ◯ | I fully agree |

11. 11) The performance of virtual visit or remote work was used by me/ my colleagues during the COVID-19 pandemic. *

*Mark only one oval.*

| | 1 | 2 | 3 | 4 | 5 | |
|---|---|---|---|---|---|---|
| I completely disagree | ◯ | ◯ | ◯ | ◯ | ◯ | I fully agree |

12. 12) I believe that virtual visit should be used much more frequently in the future. *

*Mark only one oval.*

| | 1 | 2 | 3 | 4 | 5 | |
|---|---|---|---|---|---|---|
| I completely disagree | ◯ | ◯ | ◯ | ◯ | ◯ | I fully agree |

13. 13) There was a staff shortage in my facility as a result of quarantine or infections due to the COVID-19 outbreak. *

*Mark only one oval.*

| | 1 | 2 | 3 | 4 | 5 | |
|---|---|---|---|---|---|---|
| I completely disagree | ◯ | ◯ | ◯ | ◯ | ◯ | I fully agree |

14. 14) Due to the COVID-19 pandemic, medical staff was ordered to shorten working hours, reduce overtime hours or take forced leave. *

*Mark only one oval.*

| | 1 | 2 | 3 | 4 | 5 | |
|---|---|---|---|---|---|---|
| I completely disagree | ◯ | ◯ | ◯ | ◯ | ◯ | I fully agree |

15. 15) I/ my office/ my facility received a sufficient amount of personal protective equipment (disinfectants, aprons, gloves, FFP2/ FFP3 masks) and I believe that me and my colleagues were adequately protected at work during the COVID-19 epidemic. *

*Mark only one oval.*

| | 1 | 2 | 3 | 4 | 5 | |
|---|---|---|---|---|---|---|
| I completely disagree | ◯ | ◯ | ◯ | ◯ | ◯ | I fully agree |

Which sentence listed below are true about your training course? ONLY FOR RESIDENT PHYSICIANS

16. 16) The COVID-19 pandemic reduced the number of operations I performed.

*Mark only one oval.*

| | 1 | 2 | 3 | 4 | 5 | |
|---|---|---|---|---|---|---|
| I completely disagree | ◯ | ◯ | ◯ | ◯ | ◯ | I fully agree |

17. 17) The COVID-19 pandemic has had a negative impact on the quality of education of resident physician.

*Mark only one oval.*

| | 1 | 2 | 3 | 4 | 5 | |
|---|---|---|---|---|---|---|
| I completely disagree | ◯ | ◯ | ◯ | ◯ | ◯ | I fully agree |

18. 18) The COVID-19 pandemic has increased my responsibility while working in the ward.

*Mark only one oval.*

| | 1 | 2 | 3 | 4 | 5 | |
|---|---|---|---|---|---|---|
| I completely disagree | ⬭ | ⬭ | ⬭ | ⬭ | ⬭ | I fully agree |

19. 19) The COVID-19 pandemic has extended my responsibilities as a specialist/resident physician.

*Mark only one oval.*

| | 1 | 2 | 3 | 4 | 5 | |
|---|---|---|---|---|---|---|
| I completely disagree | ⬭ | ⬭ | ⬭ | ⬭ | ⬭ | I fully agree |

20. 20) The COVID-19 pandemic had a negative impact on the result/quality of preparation for the National Specialization Examination (PES).

*Mark only one oval.*

| | 1 | 2 | 3 | 4 | 5 | |
|---|---|---|---|---|---|---|
| I completely disagree | ⬭ | ⬭ | ⬭ | ⬭ | ⬭ | I fully agree |

21. 21) The COVID-19 pandemic has adversely affected my ability to improve my surgical skills.

*Mark only one oval.*

| | 1 | 2 | 3 | 4 | 5 | |
|---|---|---|---|---|---|---|
| I completely disagree | ⬭ | ⬭ | ⬭ | ⬭ | ⬭ | I fully agree |

22. 22) The COVID-19 pandemic has reduced the number of conferences I have attended.

*Mark only one oval.*

| | 1 | 2 | 3 | 4 | 5 | |
|---|---|---|---|---|---|---|
| I completely disagree | ◯ | ◯ | ◯ | ◯ | ◯ | I fully agree |

23. 23) During the COVID-19 pandemic, I participated in online conferences.

*Mark only one oval.*

| | 1 | 2 | 3 | 4 | 5 | |
|---|---|---|---|---|---|---|
| I completely disagree | ◯ | ◯ | ◯ | ◯ | ◯ | I fully agree |

24. 24) I used webinars during the COVID-19 pandemic.

*Mark only one oval.*

| | 1 | 2 | 3 | 4 | 5 | |
|---|---|---|---|---|---|---|
| I completely disagree | ◯ | ◯ | ◯ | ◯ | ◯ | I fully agree |

25. 25) My quarantine had a negative impact on the effect of my specialization education (if relevant).

*Mark only one oval.*

| | 1 | 2 | 3 | 4 | 5 | |
|---|---|---|---|---|---|---|
| I completely disagree | ◯ | ◯ | ◯ | ◯ | ◯ | I fully agree |

26. 26) I believe that the form of online learning (conferences, webinars) positively influenced the level of my knowledge and this form of transferring knowledge should be used in the future.

*Mark only one oval.*

| | 1 | 2 | 3 | 4 | 5 | |
|---|---|---|---|---|---|---|
| I completely disagree | ◯ | ◯ | ◯ | ◯ | ◯ | I fully agree |

How COVID-19 pandemic impact on overwork and stress level?

27. 27) I feel safe doing a physical examination of a patient who is tested positive for Sars-COV-2. *

*Mark only one oval.*

| | 1 | 2 | 3 | 4 | 5 | |
|---|---|---|---|---|---|---|
| I completely disagree | ◯ | ◯ | ◯ | ◯ | ◯ | I fully agree |

28. 28) In my opinion the use of additional safety measures makes it difficult to work in the ward/ emergency room/ operating theater. *

*Mark only one oval.*

| | 1 | 2 | 3 | 4 | 5 | |
|---|---|---|---|---|---|---|
| I completely disagree | ◯ | ◯ | ◯ | ◯ | ◯ | I fully agree |

29. 29) I am afraid of Sars-COV-2 infection in the workplace. *

*Mark only one oval.*

| | 1 | 2 | 3 | 4 | 5 | |
|---|---|---|---|---|---|---|
| I completely disagree | ◯ | ◯ | ◯ | ◯ | ◯ | I fully agree |

30. 30) I feel anxious about the increased likelihood of Sars-COV-2 infection of family members. *

*Mark only one oval.*

| | 1 | 2 | 3 | 4 | 5 | |
|---|---|---|---|---|---|---|
| I completely disagree | ◯ | ◯ | ◯ | ◯ | ◯ | I fully agree |

31. 31) I have more workload during the COVID-19 pandemic than before it began. *

*Mark only one oval.*

| | 1 | 2 | 3 | 4 | 5 | |
|---|---|---|---|---|---|---|
| I completely disagree | ◯ | ◯ | ◯ | ◯ | ◯ | I fully agree |

32. 32) Due to staff shortages, there was a need to engage more work on duty. *

*Mark only one oval.*

| | 1 | 2 | 3 | 4 | 5 | |
|---|---|---|---|---|---|---|
| I completely disagree | ◯ | ◯ | ◯ | ◯ | ◯ | I fully agree |

33. 33) The SARS-CoV-2 pandemic negatively affected the atmosphere between doctors in the ward. *

*Mark only one oval.*

| | 1 | 2 | 3 | 4 | 5 | |
|---|---|---|---|---|---|---|
| I completely disagree | ◯ | ◯ | ◯ | ◯ | ◯ | I fully agree |

The COVID-19 pandemic impact on work on daily basis and patients.

34. 34) In my opinion, the number of patients hospitalized in the Orthopedic and Trauma Ward decreased by: *

*Mark only one oval.*

- 80-100 %
- 60-80%
- 40-60%
- 20-40%
- 0-20%
- It is hard to say

35. 35) In my opinion, the number of surgeries performed in the Orthopedic and Trauma Ward decreased by: *

*Mark only one oval.*

- 80-100 %
- 60-80%
- 40-60%
- 20-40%
- 0-20%
- It is hard to say

36. 36) In my opinion, the number of patients requiring immediate orthopedic assistance admitted to ICU decreased by: *

*Mark only one oval.*

- 80-100 %
- 60-80%
- 40-60%
- 20-40%
- 0-20%
- It is hard to say

37. 37) In my opinion, the number of acute (emergency) surgeries performed in the Orthopedic and Trauma Ward decreased by: *

*Mark only one oval.*

- 80-100 %
- 60-80%
- 40-60%
- 20-40%
- 0-20%
- It is hard to say

38. 38) Percentage of patients who voluntarily cancel their visit to the ward - estimate: *

*Mark only one oval.*

- 80-100 %
- 60-80%
- 40-60%
- 20-40%
- 0-20%
- It is hard to say

39. 39) Percentage number of Patients who voluntarily cancel a planned procedure - estimate: *

*Mark only one oval.*

- 80-100 %
- 60-80%
- 40-60%
- 20-40%
- 0-20%
- It is hard to say

40. 40) Due to the SARS-CoV-2 pandemic, the number of patients in my facility/ ward decreased by: *

*Mark only one oval.*

- 80-100 %
- 60-80%
- 40-60%
- 20-40%
- 0-20%
- It is hard to say

41. 41) Due to the SARS-CoV-2 pandemic, the number of patients in my private practice decreased by:

*Mark only one oval.*

- 80-100 %
- 60-80%
- 40-60%
- 20-40%
- 0-20%
- It is hard to say

Which sentence about regulations in a fight with COVID-19 pandemic are true?

42. 42) I consider myself well-informed on the latest Covid-19 regulations. *

*Mark only one oval.*

| | 1 | 2 | 3 | 4 | 5 | |
|---|---|---|---|---|---|---|
| I completely disagree | | | | | | I fully agree |

43. 43) Clinical practices conducted in Orthopedic and Trauma wards are of high importance in the fight against the SARS-CoV-2 pandemic. *

*Mark only one oval.*

| | 1 | 2 | 3 | 4 | 5 | |
|---|---|---|---|---|---|---|
| I completely disagree | ⬭ | ⬭ | ⬭ | ⬭ | ⬭ | I fully agree |

44. 44) The Polish health care system is sufficiently well prepared to fight the SARS-CoV-2 pandemic. *

*Mark only one oval.*

| | 1 | 2 | 3 | 4 | 5 | |
|---|---|---|---|---|---|---|
| I completely disagree | ⬭ | ⬭ | ⬭ | ⬭ | ⬭ | I fully agree |

45. 45) The regulations introduced so far are essential in the fight against the SARS-CoV-2 pandemic. *

*Mark only one oval.*

| | 1 | 2 | 3 | 4 | 5 | |
|---|---|---|---|---|---|---|
| I completely disagree | ⬭ | ⬭ | ⬭ | ⬭ | ⬭ | I fully agree |

46. 46) The regulations introduced so far are sufficient to fight the SARS-CoV-2 pandemic. *

*Mark only one oval.*

| | 1 | 2 | 3 | 4 | 5 | |
|---|---|---|---|---|---|---|
| I completely disagree | ⬭ | ⬭ | ⬭ | ⬭ | ⬭ | I fully agree |

47. 47) How would you rate your stress level at work before March 2020? *

*Mark only one oval.*

| 1 | 2 | 3 | 4 | 5 | 6 | 7 | 8 | 9 | 10 |
|---|---|---|---|---|---|---|---|---|---|
| ◯ | ◯ | ◯ | ◯ | ◯ | ◯ | ◯ | ◯ | ◯ | ◯ |

48. 48) How would you rate your stress level at work between March 2020 and December 2020? *

*Mark only one oval.*

| 1 | 2 | 3 | 4 | 5 | 6 | 7 | 8 | 9 | 10 |
|---|---|---|---|---|---|---|---|---|---|
| ◯ | ◯ | ◯ | ◯ | ◯ | ◯ | ◯ | ◯ | ◯ | ◯ |

What is your opinion on these prognoses about the future of pandemics?

49. 49) I think that situation on my ward will be back to normal in 2021. *

*Mark only one oval.*

| | 1 | 2 | 3 | 4 | 5 | |
|---|---|---|---|---|---|---|
| I completely disagree | ◯ | ◯ | ◯ | ◯ | ◯ | I fully agree |

50. 50) I expect to be transferred in 2021 to perform medical work outside my specialty. (eg. ICU, emergency treatment, etc). *

*Mark only one oval.*

| | 1 | 2 | 3 | 4 | 5 | |
|---|---|---|---|---|---|---|
| I completely disagree | ◯ | ◯ | ◯ | ◯ | ◯ | I fully agree |

51. 51) Me/my practise will financially suffer from COVID-19 pandemic.

*Mark only one oval.*

| | 1 | 2 | 3 | 4 | 5 | |
|---|---|---|---|---|---|---|
| I completely disagree | ◯ | ◯ | ◯ | ◯ | ◯ | I fully agree |

This content is neither created nor endorsed by Google.

Google Forms
