# Supplementary material for: The impact of the COVID-19 pandemic on Polish orthopedics, in particular on the level of stress among orthopedic surgeons and the education process
Source: PLoS One. 2021 Sep 24;16(9):e0257289. doi: 10.1371/journal.pone.0257289 (PMC8462693; doi:10.1371/journal.pone.0257289)
Supplement: S2 File — (PDF) [file pone.0257289.s003.pdf]

Wpływ pandemii COVID-19 na Ortopedię w Polsce

Anonimowa ankieta ma na celu zbadanie wpływu pandemii COVID-19 na pracę lekarzy Ortopedów, szkolenie specjalizacyjne oraz poziom stresu i przepracowania. Podzielona jest ona na 4 części. Rozwiązanie całości powinno zająć maksymalnie 10-15 minut.

Z góry serdecznie dziękujemy za poświęcony czas i wypełnienie poniższej ankiety

Jestem świadom, że udział w ankiecie jest dobrowolny i anonimowy oraz zgadzam się na udział w badaniu poprzez przejście do kolejnej części ankiety

*Wymagane

W tej części ankiety chcielibyśmy dowiedzieć się podstawowych informacji dotyczących Państwa.

1. 1) Jaka jest Twoja płeć? *

Zaznacz tylko jedną odpowiedź.

- Kobieta
- Mężczyzna

2. 2) Do jakiej grupy wiekowej się kwalifikujesz? *

Zaznacz tylko jedną odpowiedź.

- < 35 lat
- 35-49 lat
- 50-69 lat
- > 70 lat

3. 3) Ile lat pracujesz jako ortopeda (wliczając okres specjalizacji)? *

Zaznacz tylko jedną odpowiedź.

- 0-5 lat
- 6-10 lat
- 11-20 lat
- >20 lat

4. 4) Jakie stanowisko zajmujesz obecnie w swoim głównym miejscu pracy? *

Zaznacz tylko jedną odpowiedź.

- Rezydent
- Lekarz specjalista
- Ordynator\zastępca Ordynatora
- Samozatrudnienie w praktyce prywatnej
- Inne

5. 5) Główny obszar pracy i zainteresowań: (możliwa więcej niż jedna odpowiedź; max 3 odpowiedzi) *

Zaznacz wszystkie właściwe odpowiedzi.

- Ortopedia ogólna
- Traumatologia
- Onkologia Narządu Ruchu
- Artroplastyka stawów
- Artroskopia i ortopedia małoinwazyjna
- Ortopedia kręgosłupa
- Ortopedia pediatryczna
- Chirurgia ręki
- Chirurgia w obrębie stopy
- Inne

6. 6) W jakiej instytucji w ramach pracy jako lekarz ortopeda wykonujesz obecnie (większość) swoją pracę? *

Zaznacz tylko jedną odpowiedź.

- Szpital Uniwersytecki/Szpital o najwyższym stopniu referencyjności
- Szpital powiatowy
- Prywatna praktyka
- Inne

Jak pandemia COVID-19 wpłynęła na Państwa gabinet/ klinikę/ oddział?

7. 7) Mój gabinet/ moja klinika/ ja jestem aktualnie zaangażowany w leczenie pacjentów COVID-19 dodatnich. *

Zaznacz tylko jedną odpowiedź.

	1	2	3	4	5	
W pełni się nie zgadzam						W pełni się zgadzam

8. 8) Mój gabinet/ moja klinika/ ja zastosowałem środki ochrony oraz zmianę organizacji pracy by przeciwdziałać rozprzestrzenianiu się COVID-19. *

Zaznacz tylko jedną odpowiedź.

	1	2	3	4	5	
W pełni się nie zgadzam						W pełni się zgadzam

9. 9) W mojej jednostce, personel został podzielony w mniejsze grupy, by zminimalizować ryzyko zakażenia. *

Zaznacz tylko jedną odpowiedź.

	1	2	3	4	5	
W pełni się nie zgadzam	( )	( )	( )	( )	( )	W pełni się zgadzam

10. 10) W mojej jednostce, zarówno pacjenci zdrowi jak i zarażeni byli izolowani w oddzielnych pomieszczeniach. *

Zaznacz tylko jedną odpowiedź.

	1	2	3	4	5	
W pełni się nie zgadzam	( )	( )	( )	( )	( )	W pełni się zgadzam

11. 11) Wykonywanie teleporad lub pracy zdalnej zostało przeze mnie/ moich kolegów użyte podczas pandemii COVID-19. *

Zaznacz tylko jedną odpowiedź.

	1	2	3	4	5	
W pełni się nie zgadzam	( )	( )	( )	( )	( )	W pełni się zgadzam

12. 12) Uważam, że teleporady powinny w przyszłości być stosowane zdecydowanie częściej. *

Zaznacz tylko jedną odpowiedź.

	1	2	3	4	5	
W pełni się nie zgadzam	( )	( )	( )	( )	( )	W pełni się zgadzam

13. 13) W wyniku kwarantanny lub zakażeń z powodu epidemii COVID-19 w mojej placówce widoczne były braki personelu. *

Zaznacz tylko jedną odpowiedź.

	1	2	3	4	5	
W pełni się nie zgadzam	◯	◯	◯	◯	◯	W pełni się zgadzam

14. 14) Z powodu pandemii COVID-19 pracownikom kazano skrócić godziny pracy, zmniejszyć ilość nadgodzin lub brać przymusowy urlop. *

Zaznacz tylko jedną odpowiedź.

	1	2	3	4	5	
W pełni się nie zgadzam	◯	◯	◯	◯	◯	W pełni się zgadzam

15. 15) Ja/mój gabinet/moja placówka otrzymaliśmy wystarczającą ilość środków ochrony osobistej (środki do dezynfekcji, fartuchy, rękawiczki, maski FFP2/FFP3) oraz uważam że ja oraz moi współpracownicy byliśmy odpowiednio chronieni w pracy podczas epidemii COVID-19. *

Zaznacz tylko jedną odpowiedź.

	1	2	3	4	5	
W pełni się nie zgadzam	◯	◯	◯	◯	◯	W pełni się zgadzam

Które z poniższych zdań na temat szkolenia rezydenckiego dotyczy Państwa sytuacji? (TYLKO DLA LEKARZY PODCZAS SPECJALIZACJI)

16. 16) Pandemia COVID-19 zmniejszyła ilość wykonywanych przeze mnie zabiegów.

Zaznacz tylko jedną odpowiedź.

	1	2	3	4	5	
W pełni się nie zgadzam	◯	◯	◯	◯	◯	W pełni się zgadzam

17. 17) Pandemia COVID-19 wpłynęła negatywnie na jakość kształcenia lekarzy rezydentów.

Zaznacz tylko jedną odpowiedź.

	1	2	3	4	5	
W pełni się nie zgadzam	◯	◯	◯	◯	◯	W pełni się zgadzam

18. 18) Pandemia COVID-19 spowodowała zwiększenie ponoszenia przeze mnie odpowiedzialności w trakcie pracy na oddziale.

Zaznacz tylko jedną odpowiedź.

	1	2	3	4	5	
W pełni się nie zgadzam	◯	◯	◯	◯	◯	W pełni się zgadzam

19. 19) Pandemia COVID-19 wpłynęła na poszerzenie zakresu moich obowiązków jako lekarza specjalisty/rezydenta.

Zaznacz tylko jedną odpowiedź.

	1	2	3	4	5	
W pełni się nie zgadzam	◯	◯	◯	◯	◯	W pełni się zgadzam

20. 20) Pandemia COVID-19 wpłynęła negatywnie na wynik/jakość przygotowania do Państwowego Egzaminu Specjalizacyjnego.

Zaznacz tylko jedną odpowiedź.

	1	2	3	4	5	
W pełni się nie zgadzam	◯	◯	◯	◯	◯	W pełni się zgadzam

21. 21) Pandemia COVID-19 wpłynęła negatywnie na możliwość doskonalenia moich umiejętności zabiegowych.

Zaznacz tylko jedną odpowiedź.

	1	2	3	4	5	
W pełni się nie zgadzam	◯	◯	◯	◯	◯	W pełni się zgadzam

22. 22) Pandemia COVID-19 zmniejszyła ilość konferencji w których uczestniczyłem.

Zaznacz tylko jedną odpowiedź.

	1	2	3	4	5	
W pełni się nie zgadzam	◯	◯	◯	◯	◯	W pełni się zgadzam

23. 23) Podczas pandemii COVID-19 brałem udział w konferencjach online.

Zaznacz tylko jedną odpowiedź.

	1	2	3	4	5	
W pełni się nie zgadzam	◯	◯	◯	◯	◯	W pełni się zgadzam

24. 24) Podczas pandemii COVID-19 korzystałem z webinarów.

Zaznacz tylko jedną odpowiedź.

	1	2	3	4	5	
W pełni się nie zgadzam	◯	◯	◯	◯	◯	W pełni się zgadzam

25. 25) Odbywanie przeze mnie kwarantanny wpłynęło negatywnie na efekt mojego kształcenia specjalizacyjnego (jeśli dotyczy)

Zaznacz tylko jedną odpowiedź.

	1	2	3	4	5	
W pełni się nie zgadzam	◯	◯	◯	◯	◯	W pełni się zgadzam

26. 26) Uważam, że forma nauki online (konferencje, webinary) pozytywnie wpłynęły na poziom mojej wiedzy i taka forma przekazu wiedzy powinna być stosowana w przyszłości.

Zaznacz tylko jedną odpowiedź.

	1	2	3	4	5	
W pełni się nie zgadzam	◯	◯	◯	◯	◯	W pełni się zgadzam

Jak Pandemia wpłynęła na poziom przepracowania i stresu w Państwa przypadku?

27. 27) Czuję się bezpiecznie wykonując badanie fizykalne pacjenta, z dodatnim wynikiem testu w kierunku wirusa Sars-COV-2. *

Zaznacz tylko jedną odpowiedź.

	1	2	3	4	5	
W pełni się nie zgadzam	◯	◯	◯	◯	◯	W pełni się zgadzam

28. 28) Stosowanie dodatkowych środków bezpieczeństwa epidemiologicznego utrudnia, moim zdaniem pracę na oddziale/izbie przyjęć/bloku operacyjnym. *

Zaznacz tylko jedną odpowiedź.

	1	2	3	4	5	
W pełni się nie zgadzam	⬭	⬭	⬭	⬭	⬭	W pełni się zgadzam

29. 29) Odczuwam obawę przed zakażeniem wirusem Sars-COV-2 w miejscu pracy. *

Zaznacz tylko jedną odpowiedź.

	1	2	3	4	5	
W pełni się nie zgadzam	⬭	⬭	⬭	⬭	⬭	W pełni się zgadzam

30. 30) Odczuwam lęk związany ze zwiększonym prawdopodobieństwem zarażenia członków rodziny wirusem Sars-COV-2. *

Zaznacz tylko jedną odpowiedź.

	1	2	3	4	5	
W pełni się nie zgadzam	⬭	⬭	⬭	⬭	⬭	W pełni się zgadzam

31. 31) Odczuwam większe obciążenie pracą w trakcie trwania pandemii COVID-19 niż przed jej rozpoczęciem. *

Zaznacz tylko jedną odpowiedź.

	1	2	3	4	5	
W pełni się nie zgadzam	⬭	⬭	⬭	⬭	⬭	W pełni się zgadzam

32. 32) Z powodów braków kadrowych była potrzeba podejmować więcej pracy w trybie dyżurowym. *

Zaznacz tylko jedną odpowiedź.

	1	2	3	4	5	
W pełni się nie zgadzam						W pełni się zgadzam

33. 33) Pandemia związana z SARS-CoV-2 wpłynęła negatywnie na atmosferę miedzy lekarzami na oddziale. *

Zaznacz tylko jedną odpowiedź.

	1	2	3	4	5	
W pełni się nie zgadzam						W pełni się zgadzam

Wpływ pandemii SARS- CoV-2 na codzienną pracę/ pacjentów.

34. 34) W mojej ocenie, liczba pacjentów poddawanych hospitalizacji na Oddziale Ortopedyczno-Urazowym zmniejszyła się o: *

Zaznacz tylko jedną odpowiedź.

- 80-100 %
- 60-80%
- 40-60%
- 20-40%
- 0-20%
- Trudno określić

35. 35) W mojej ocenie, liczba operacji planowych wykonywanych na Oddziale Ortopedyczno-Urazowym zmniejszyła się o: *

Zaznacz tylko jedną odpowiedź.

- 80-100 %
- 60-80%
- 40-60%
- 20-40%
- 0-20%
- Trudno określić

36. 36) W mojej ocenie, liczba pacjentów wymagających natychmiastowej pomocy ortopedycznej, przyjmowanych na SOR zmniejszyła się o: *

Zaznacz tylko jedną odpowiedź.

- 80-100 %
- 60-80%
- 40-60%
- 20-40%
- 0-20%
- Trudno określić

37. 37) W mojej ocenie liczba wykonywanych operacji ostrych (nagłych) na Oddziale Ortopedyczno-Urazowym zmniejszyła się o: *

Zaznacz tylko jedną odpowiedź.

- 80-100 %
- 60-80%
- 40-60%
- 20-40%
- 0-20%
- Trudno określić

38. 38) Procentowo liczba Pacjentów, którzy z własnej woli odwołują wizytę na oddziale – wyraź szacunkowo *

Zaznacz tylko jedną odpowiedź.

- 80-100 %
- 60-80%
- 40-60%
- 20-40%
- 0-20%
- Trudno określić

39. 39) Procentowo liczba Pacjentów, którzy z własnej woli odwołują zaplanowany zabieg – wyraź szacunkowo *

Zaznacz tylko jedną odpowiedź.

- 80-100 %
- 60-80%
- 40-60%
- 20-40%
- 0-20%
- Trudno określić

40. 40) W związku z pandemią SARS- CoV-2 liczba pacjentów w mojej placówce/ na oddziale zmniejszyła się o: *

Zaznacz tylko jedną odpowiedź.

- 80-100 %
- 60-80%
- 40-60%
- 20-40%
- 0-20%
- Trudno określić

41. 41) W związku z pandemią SARS- CoV-2 liczba pacjentów w mojej praktyce prywatnej zmniejszyła się o:

Zaznacz tylko jedną odpowiedź.

- 80-100 %
- 60-80%
- 40-60%
- 20-40%
- 0-20%
- Trudno określić

Zaznacz, w jakim stopniu zgadzasz się z twierdzeniami dotyczącymi środków podjętych w walce z epidemią SARS-CoV-2.

42. 42) Uważam się za dobrze poinformowanego w kwestii najnowszych regulacji dotyczących Covid-19. *

Zaznacz tylko jedną odpowiedź.

	1	2	3	4	5	
W pełni się nie zgadzam						W pełni się zgadzam

43. 43) Praktyki kliniczne prowadzone na oddziałach Ortopedyczno-Urazowych mają wysokie znaczenie w walce z pandemią SARS-CoV-2 *

Zaznacz tylko jedną odpowiedź.

	1	2	3	4	5	
W pełni się nie zgadzam						W pełni się zgadzam

44. 44) Polska służba zdrowia jest wystarczająco dobrze przygotowana do walki z pandemia SARS-CoV-2 *

Zaznacz tylko jedną odpowiedź.

	1	2	3	4	5	
W pełni się nie zgadzam	⬭	⬭	⬭	⬭	⬭	W pełni się zgadzam

45. 45) Regulacje wprowadzone do tej pory są niezbędne w walce z pandemią SARS-CoV-2. *

Zaznacz tylko jedną odpowiedź.

	1	2	3	4	5	
W pełni się nie zgadzam	⬭	⬭	⬭	⬭	⬭	W pełni się zgadzam

46. 46) Regulacje wprowadzone do tej pory są wystarczające do walki z pandemia SARS-CoV-2 *

Zaznacz tylko jedną odpowiedź.

	1	2	3	4	5	
W pełni się nie zgadzam	⬭	⬭	⬭	⬭	⬭	W pełni się zgadzam

47. 47) Jak oceniłbyś swój poziom stresu w pracy przed Marcem 2020? *

Zaznacz tylko jedną odpowiedź.

1	2	3	4	5	6	7	8	9	10
⬭	⬭	⬭	⬭	⬭	⬭	⬭	⬭	⬭	⬭

48. 48) Jak oceniłbyś swój poziom stresu w pracy w okresie Marzec 2020 – Grudzień 2020? *

Zaznacz tylko jedną odpowiedź.

1	2	3	4	5	6	7	8	9	10
⬭	⬭	⬭	⬭	⬭	⬭	⬭	⬭	⬭	⬭

Jak reagujesz na poniższe prognozy dotyczące nadchodzących wydarzeń związanych z pandemią COVID-19?

49. 49) Przewiduję, że tryb pracy w mojej placówce zacznie ulegać normalizacji od 2021r. *

Zaznacz tylko jedną odpowiedź.

	1	2	3	4	5	
W pełni się nie zgadzam	⬭	⬭	⬭	⬭	⬭	W pełni się zgadzam

50. 50) Oczekuję, że zostanę przeniesiony w 2021r. do wykonywania pracy medycznej poza własną specjalnością (np. Oddział intensywnej terapii, leczenie pacjentów wentylowanych,emergency treatment, ...) *

Zaznacz tylko jedną odpowiedź.

	1	2	3	4	5	
W pełni się nie zgadzam	⬭	⬭	⬭	⬭	⬭	W pełni się zgadzam

51. 51) Ja/moja praktyka prywatna/moja klinika napotkam trudności finansowe z powodu pandemii COVID-19.

Zaznacz tylko jedną odpowiedź.

	1	2	3	4	5	
W pełni się nie zgadzam	⬭	⬭	⬭	⬭	⬭	W pełni się zgadzam

Ta treść nie została utworzona ani zatwierdzona przez Google.

Formularze Google
